# Inhaled or Ingested, Which Is Worse, E-Vaping or High-Fat Diet?

**DOI:** 10.3389/fimmu.2022.913044

**Published:** 2022-06-15

**Authors:** Hui Chen, Yik Lung Chan, Andrew E. Thorpe, Carol A. Pollock, Sonia Saad, Brian G. Oliver

**Affiliations:** ^1^ School of Life Sciences, Faculty of Science, University of Technology Sydney, Sydney, NSW, Australia; ^2^ Respiratory Cellular and Molecular Biology, Woolcock Institute of Medical Research, The University of Sydney, Sydney, NSW, Australia; ^3^ Kolling Institute of Medical Research, Royal North Shore Hospital, The University of Sydney, Sydney, NSW, Australia

**Keywords:** e-cigarette, inflammatory cytokines, serum, obesity, nicotine free

## Abstract

Long term e-cigarette vaping induces inflammation, which is largely nicotine independent. High-fat diet (HFD) consumption is anoter cause of systemic low-grade inflammation. The likelihood of using e-cigarettes as a weight control strategy is concomitant with the increase in obesity. In Australia, only nicotine-free e-fluid is legal for sale. Therefore, this study aimed to investigate how nicotine-free e-cigarette vapour exposure affects inflammatory responses in mice with long term HFD consumption. Mice were fed a HFD for 16 weeks, while in the last 6 weeks, half of the chow and HFD groups were exposed to nicotine-free e-vapour, while the other half to ambient air. Serum, lung, liver and epididymal fat were collected to measure inflammatory markers. While both e-vapour exposure and HFD consumption independently increased serum IFN-γ, CX3CL1, IL-10, CCL20, CCL12, and CCL5 levels, the levels of IFN-γ, CX3CL1, and IL-10 were higher in mice exposed to e-vapour than HFD. The mRNA expression pattern in the epididymal fat mirrors that in the serum, suggesting the circulating inflammatory response to e-vapour is from the fat tissue. Of the upregulated cytokines in serum, none were found to change in the lungs. The anti-inflammatory cytokine IL-10 was increased by combining e-vapour and HFD in the liver. We conclude that short-term nicotine-free e-vapour is more potent than long term HFD consumption in causing systemic inflammation. Future studies will be needed to examine the long-term health impact of nicotine-free e-cigarettes.

## Introduction

Obesity is characterised by excessive accumulation of adipose tissue, often caused by overconsumption of a diet high in energy and fat (HFD) and insufficient physical activities to consume the additional energy intake (1). The lipid influx resulting from a HFD consumption leads to a low-grade inflammatory status, due to the recruitment and accumulation of tissue macrophages even before the onset of obesity (2, 3). This further leads to a number of comorbidities, including cerebrovascular and cardiovascular diseases due to the development of atherosclerosis, diabetes due to systemic insulin resistance, and increased risk of asthma (1, 4). Tobacco cigarette smoking has been popular particularly among young people, to manage appetite and body weight, which also prevented them from quitting due to the concerns of significant weight gain afterwards (5). With the rising popularity of e-cigarettes, they have also been used for the same purpose (6–8). Indeed, a recent study found that obese individuals are more likely to use e-cigarette for weight loss purposes, more among men than women (9), as well as among those with eating disorders (10), even though, there has been no evidence to suggest that this approach is effective.

E-cigarettes are marketed to assist with smoking cessation and are frequently described as a ‘safe cigarette’ with fewer toxicants and lower risks of diseases. However, in recent years, e-cigarettes have gained significant popularity amongst younger people, albeit the latest report on acute lung injury and death in the US (11, 12). While nicotine-containing e-cigarettes have been used to satisfy a nicotine addiction, even among those who never used tobacco cigarettes, nicotine-free e-cigarettes have also been largely used recreationally by ‘cloud chasers’ (13).

The recognition of the harmful effects of e-cigarettes is increasing among adults (14); however, there is a common misconception that only nicotine is harmful (15). What is more worrying is that more teenagers have started to vape because they think that e-cigarettes are harmless (16). In the US, only the sale of tobacco-flavoured e-fluids is permitted to reduce the potential harm induced by the flavouring chemicals. However, in Australia, only the sale of nicotine-free e-fluid is legal. We previously showed that in a mouse model of e-cigarette vaping, exposure to nicotine-free e-cigarette vapour for 12 weeks can increase inflammation in the lung and liver (17, 18). It is now well acknowledged that chronic inflammation is a common pathway involved in the pathogenesis of disorders of multiple organs, which can occur at any stage of life (19). Given that the use of e-cigarettes to control obesity resulting from excessive caloric intake, such as a HFD, has become a norm in society (6, 8), here in a mouse model of HFD consumption induced adiposity, we aimed to investigate whether nicotine-free e-vapour inhalation interacts with overconsumption of a high calorie and high fat diet (HFD) to affect the inflammatory profile in multiple systems (including the serum, lung, liver and fat tissue) that contribute to the overall systemic inflammatory status. The brain was not included in this study due to the lack of inflammatory response in our previous study (20).

## Material and Methods

### Modelling Obesity and E-Vapour Exposure

The animal experiments were approved by the Animal Ethics and Care Committee at Northern Sydney Health District (RESP17/93) and published previously (20). Briefly, male Balb/c mice (7 weeks) were fed a HFD (43% energy from fat (canola oil 5g/100g, cocoa butter 5g/100g, hydrogenated vegetable oil 13.1g/100g), 17% energy from protein, 40% energy from carbohydrate, 20kJ/g, Specialty Feeds, WA, Australia) for 10 weeks to induce obesity with standard chow as control (17% energy from fat (saturated 1.49g/100g, monounsaturated 1.81g/100g, polyunsaturated ω6 2.2g/100g, polyunsaturated ω3 0.65g/100g), 29.7% energy from protein, 53% energy from carbohydrate, 13kJ/g, Gordon’s Specialty Stockfeeds, NSW, Australia). From weeks 11-16, two sub-groups of mice in each dietary group were exposed to nicotine-free e-vapour (0mg/mL, tobacco flavour, 50% Propylene Glycol/50%Vegetable Glycerin,Vaper Empire, VIC) or ambient air (Sham) for 30 minutes, twice daily for 6 weeks in a 19L chamber as we have previously published; while the same diets were maintained. This generated four experimental groups: Chow+sham, Chow+e-vapour, HFD+sham, and HFD+e-vapour. The last e-vapour was administered at 3 pm, and mice were sacrificed at 8 am the following morning. At the endpoint, after deep anaesthesia with isoflurane (2%), blood was collected *via* cardiac puncture and chemokines in the serum were measured by a Bio-Plex Pro™ Mouse Chemokine Panel 33-Plex kit (Bio-Rad) according to the manufacturer’s instruction. Lungs, livers and epididymal fat were harvested, snap frozen and stored at -80°C for RNA extraction.

### Qualitative Real-Time PCR

**Figure 1:**
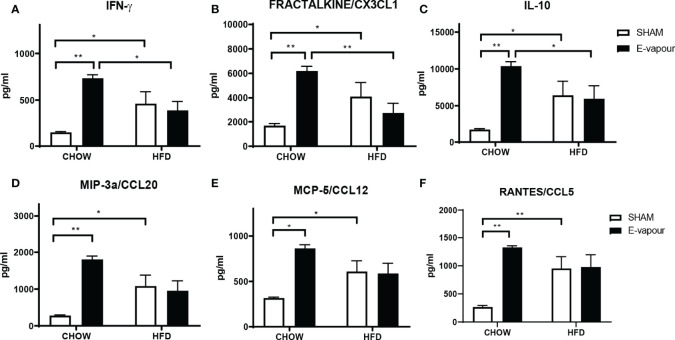
Serum levels of cytokines IFN-γ **(A)**, FRACTALKINE/CX3CL1 **(B)**, IL-10 **(C)**, MIP-3a/CCL20 **(D)**, MCP-5/CCL12 **(E)**, and RANTES/CCL5 **(F)**. Results are expressed as mean ± SEM, n = 5. *P < 0.05, **P < 0.01.

Based on the above serum Chemokine Panel assay results, 6 genes known to be important in e-vaping and obesity were selected for measurement in the lung, liver, and fat tissue to compare the serum cytokine levels (**Figure 1**). Total mRNA was extracted from lungs, livers and retroperitoneal fat using TriZol reagent (Sigma). Purified mRNA was used as a template to generate first-strand cDNA using M-MLV Reverse Transcriptase, RNase H, Point Mutant Kit (Promega, Madison, WI, USA). Target genes were measured using manufactured pre-designed KiCqStart**^®^** SYBR Green Primers **^®^**. Before acquiring the actual data, all the new primers were tested for specificity (Single peak in dissociation curve analysis). Gene expression was standardised to β actin expression. Primers sequences were provided in **Supplementary Table 1**. The average expression of the control group was assigned as the calibrator against which all other samples are expressed as fold difference.

### Statistical Methods

Results are expressed as Mean ± standard error of the mean (SEM) and analysed by two-ANOVA, followed by Tukey *post hoc* tests (GraphPad Prism 9.2, GraphPad, CA, USA). P<0.05 is considered significant.

## Results

### Serum Cytokine Levels

HFD consumption and e-vapour exposure independently increased the levels of all 6 major cytokines of interest, including IFN-γ (HFD P<0.05, e-vapour P<0.01, vs Chow+sham, **Figure 1A**), FRACTALKINE/CX3CL1 (HFD P<0.05, e-vapour P<0.01, vs Chow+sham, **Figure 1B**), IL-10 (HFD P<0.05, e-vapour P<0.01, vs Chow+sham, **Figure 1C**), MIP-3a/CCL20 (HFD P<0.05, e-vapour P<0.01, vs Chow+sham, **Figure 1D**), MCP-5/CCL12 (HFD P<0.05, e-vapour P<0.05, vs Chow+sham, **Figure 1E**), and RANTES/CCL5 (HFD P<0.01, e-vapour P<0.01, vs Chow+sham, **Figure 1F**). The levels of IFN-γ (P<0.05, **Figure 1A**), FRACTALKINE/CX3CL1 (P<0.01, **Figure 1B**), and IL-10 (P<0.05, **Figure 1C**) levels were higher in the Chow+e-vapour group compared with the HFD+e-vapour group. There were no additive effects between e-vapour exposure and HFD consumption.

Serum levels of TNFα, MPI-1β and MIP-3α were increased by HFD and e-vapour exposure alone (P<0.05 Chow+sham vs Chow+e-vapour and Chow+sham vs HFD+sham, **Supplementary Table 2**) which were expected, but not the combination of HFD and e-vapour exposure. ENA-78, IL-4, IP-10, and MCP-5 levels were only increased by e-vapour exposure (P<0.05 for ENA-78, IL-4, and IP-10; P<0.01 for MCP-5 vs Chow+sham, **Supplementary Table 2**). The MIP-3β level was only increased by HFD (P<0.05, vs Chow-sham). The other cytokines measured in this study include IL-1β, IL-16, MIP-1, I-309, MCP-3, MDC, KC, TARC, SDF-1A, EOTAXIN-1, and EOTAXIN-2, which were not significantly changed (**Supplementary Table 2**).

### mRNA Expression

**Figure 2:**
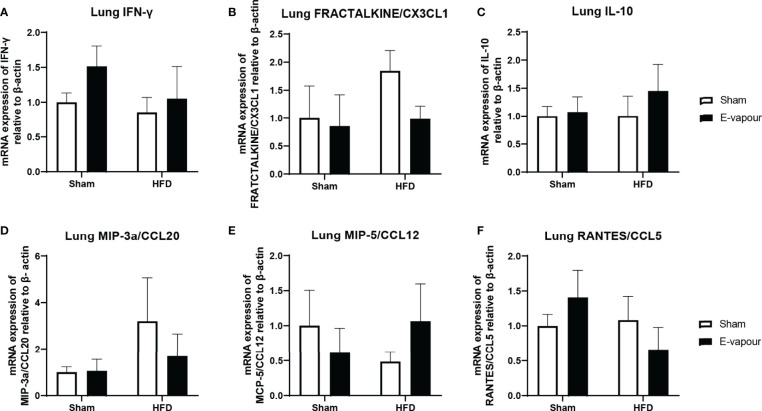
mRNA expression of IFN-γ **(A)**, FRACTALKINE/CX3CL1 **(B)**, IL-10 **(C)**, MIP-3a/CCL20 **(D)**, MCP-5/CCL12 **(E)**, and RANTES/CCL5 **(F)** in the lung. Results are expressed as mean ± SEM, n=5.

**Figure 3:**
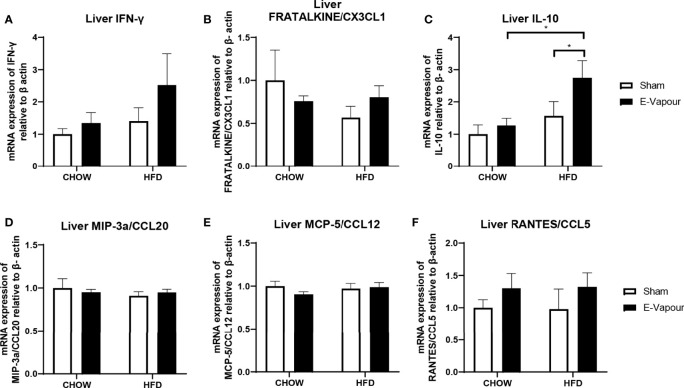
mRNA expression of IFN-γ **(A)**, FRACTALKINE/CX3CL1 **(B)**, IL-10 **(C)**, MIP-3a/CCL20 **(D)**, MCP-5/CCL12 **(E)**, and RANTES/CCL5 **(F)** in the liver. Results are expressed as mean ± SEM, n=5-7. *P < 0.05.

In the lung, mRNA expression of IFN-γ, FRACTALKINE/CX3CL1, IL-10, MIP-3a/CCL20, MCP-5/CCL12 and RANTES/CCL5 was not significantly changed by either intervention (**Figure 2**). In the liver, mRNA levels of IFN-γ (**Figure 3A**) and IL-10 (**Figure 3C**) were increased by the combination of HFD consumption and e-vapour exposure (HFD+e-vapour group); however, only IL-10 reached statistical significance (P<0.05 HFD+e-vapour vs HFD+sham and Chow+e-vapour). FEACTALKINE/CX3CL1, MIP-3a/CCL20, MCP-5/CCL12 and RANTES/CCL5 mRNA expression was not different between groups (**Figure 3**).

**Figure 4:**
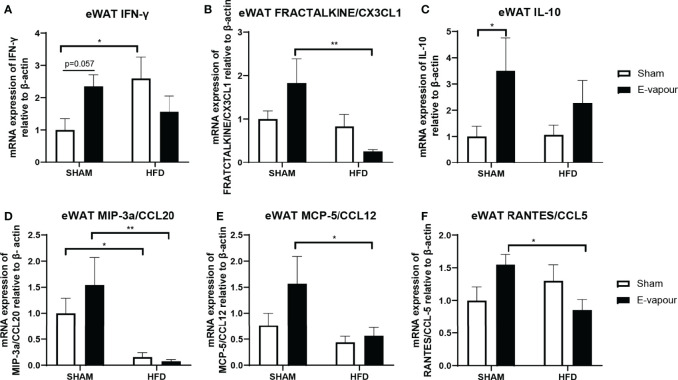
mRNA expression of IFN-γ **(A)**, FRACTALKINE/CX3CL1 **(B)**, IL-10 **(C)**, MIP-3a/CCL20 **(D)**, MCP-5/CCL12 **(E)**, and RANTES/CCL5 **(F)** in epididymal white adipose tissue (eWAT). Results are expressed as mean ± SEM, n=7-8. *P < 0.05, **P < 0.01.

In the epididymal white adipose tissue, HFD consumption increases the mRNA level of IFN-γ (P<0.05 HFD+sham vs Chow+sham, **Figure 4A**). HFD also significantly downregulated mRNA level of MIP-3a/CCL20 compared to Chow-fed mice (P<0.05, **Figure 4D**). E-vapour increased IFN-γ mRNA expression (P=0.057, **Figure 4A**) and IL-10 mRNA expression (P<0.05,**Figure 4C**). Interestingly, the combination of HFD consumption and e-vapour exposure suppressed the response of FEACTALKINE/CX3CL1 (P<0.01 Chow+e-vapour vs HFD+e-vapour, **Figure 4B**), MIP-3a/CCL20 (P<0.01 Chow+e-vapour vs HFD+e-vapour, **Figure 4D**), MCP-5/CCL12 (P<0.05 Chow+e-vapour vs HFD+e-vapour, **Figure 4E**), and RANTES/CCL5 (P<0.05 Chow+e-vapour vs HFD+e-vapour, **Figure 4F**) due to e-vapour exposure alone.

## Discussion

The major finding in this study is that nicotine-free e-vapour exposure alone can elicit a strong systemic inflammatory response in the serum and abdominal adipose tissue, even more so than HFD-consumption alone. However, the addition of HFD consumption suppressed such inflammatory responses in the serum and adipose tissue. Therefore, adipose tissue, but not the lung, may be the major driver of inflammation in response to nicotine-free e-vapour exposure alone; however, unknown mechanisms may contribute to the suppressed inflammatory effects in the serum and adipose tissue observed with the combination of HFD and nicotine-free e-vapour.

A panel of inflammatory cytokines was increased by e-vapour exposure. Among those, the increase in circulating IFN-γ was previously found in the serum of e-cigarette users (21). However, the human study focused on nicotine e-cigarette users, whereas the current study was on nicotine-free e-vapour. IFN-γ activates macrophages, natural killer cells and neutrophils, which in turn stimulates the release of downstream CX3CL1, CCL20, CCL12 and CCL5 (22). We also observed several increased pro-inflammatory cytokines by chronic HFD consumption alone, such as TNF-α, MPI-1β, MIP-3α and MIP-3β. Previously studies using C57BL/6 mice found increased serum IL-6, but not TNFα or MCP-1 (23, 24). In the study by Catta-Preta and colleagues, male mice fed a HFD (22.7 kJ/g) with 60% energy from fat for 10 weeks; while in the study by Ludgero-Correia and colleagues, female mice were fed a similar HFD for 18 weeks (23, 24). In both studies, the standard chow only contains 10% energy from the fat, whereas our standard chow has approximately double energy from fat. Therefore the difference in fat composition between HFD and the control group, as well as the use of different mice strains, may explain the difference between our study and those by Catta-Preta et al. and Ludgero-Correia et al.

We subsequently examined the changes in different organs that may contribute to the serum changes. As the first and direct target for e-vapour exposure, lungs were first examined. The lack of lung response was unexpected in the e-vapour groups. The mRNA expression of all six genes, IFN-γ, FRACTALKINE/CX3CL1, IL-10, MIP-3a/CCL20, MCP-5/CCL12, and RANTES/CCL5, was not significantly changed by e-vapour exposure. Our finding in the lung is consistent with a previous study. In this study, mice exposed to nicotine-free e-cigarette vapour for 4 months did not develop pulmonary inflammation or emphysema (25). However, lipid homeostasis was altered in alveolar macrophages and epithelial cells, which made mice more susceptible to influenza infection (25). As there is a lack of human data exploring lung pathology in response to nicotine-free e-cigarette use, we assume that without additional insults, such as respiratory infection, the influence on lung pathology and physiology can be minor by sub-chronic daily exposure in the short term, which may make users feel safe to continue to vape nicotine-free e-fluid. However, the serum cytokine changes advise differently, suggesting vaping nicotine-free e-fluid is not without adverse health impacts.

The polarisation of macrophages to a “M1” pro-inflammatory status by upregulation of IFN-γ due to vaping is consistent with prior studies, which suggest M1 macrophages play a major role in metabolic diseases driven by dysregulated adipose tissue function (22). Upon exposure to nicotine-free e-vapour alone, IFN-γ mRNA was significantly increased with an adaptive increase in IL-10 mRNA levels in the adipose tissue, which may not be sufficient to counteract the enhanced inflammatory response locally. Adipose tissue is not just the storage of excess energy from our diet, but is also considered an independent organ with significant physiological functions (26). The excess nutrient influx into the adipocytes and the resulting increase in lipolysis and activation of adipose tissue resident macrophages attract more monocytes from the circulation to become adipose tissue macrophages (27). This can further increase IFN-γ production from monocytes. Without exogenous pathogen infection, these adipose tissue macrophages are the major source of chronic systemic inflammation in obesity (26), by activating NF-κb signalling (28). Such chronic low-grade systemic inflammation can cause various metabolic disorders in major organ systems (19). As expected, we found that HFD consumption can increase serum levels of several pro-inflammatory cytokines, with an adaptation increase in IL-10 levels. Chemotactic protein-5 (MCP-5/CCL12) can be used as an M1 macrophage marker (29), and RANTES/CCL5 is secreted by macrophages (30). It seems that in our model, adipose tissue macrophage infiltration may only be increased by e-vapour alone, whereas HFD increases the secretion activity of adipose tissue macrophages. As adipose tissue macrophages play a key role in the systemic low-grade inflammatory status induced by HFD consumption, IL-6 and TNFα are the most commonly measured inflammatory markers in both blood and metabolic organs in the literature. Serum IL-6 was undetectable in the Balb/c mice used in this study, whereas serum TNFα levels were increased by HFD consumption, which is normally observed in adipose tissue by us and the others (24, 31, 32).

**Figure 5:**
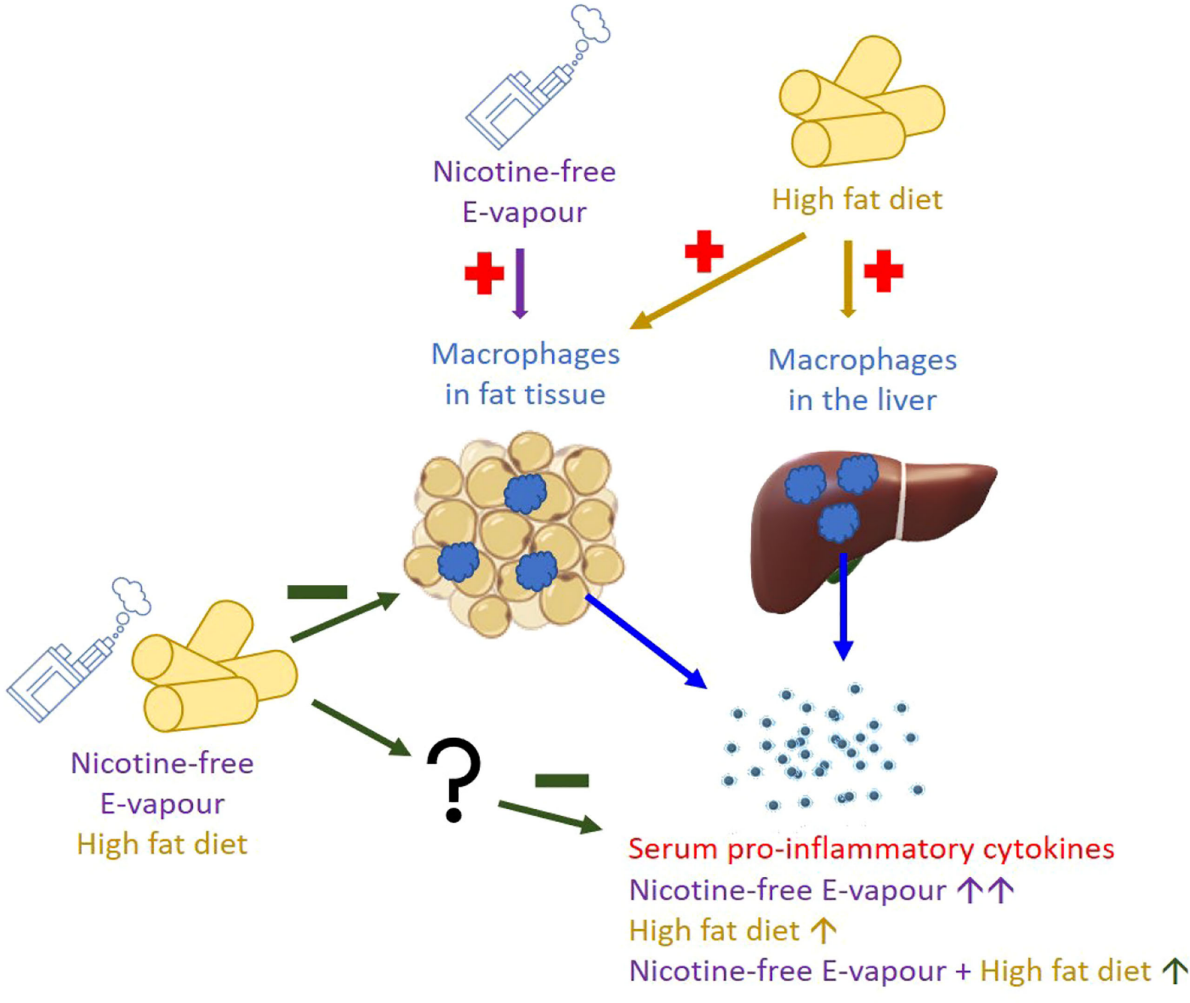
Proposed working mechanism.

As nicotine is known to suppress appetite and smoking cessation is often accompanied by significant weight gain, tobacco cigarettes used to be a popular option for weight control 33). After e-cigarettes gained popularity, overweight individuals are more likely to vape nicotine containing e-fluids, aiming to lose or control their body weights (6, 7), including adolescents (8). On the other hand, nicotine-free e-cigarettes have also been used by those who never smoked and ex-smokers who have quit tobacco by using nicotine-containing e-cigarettes as a ‘cloud chaser’ or “vaper” (13. However, the health impacts of nicotine-free e-cigarettes are less known compared to nicotine-containing e-cigarettes, which have been studied in both human and animal models. In this study, the combination of HFD and e-vapour suppressed several genes coding pro-inflammatory cytokines downstream of IFN-γ that were increased in the Chow+e-vapour group. We first suspected that this may be through the upregulated anti-inflammatory IL-10 mRNA in the liver, the only marker known to support such an effect. Although the increase in IFN-γ by the combination of HFD and e-vapour was not significant, in our previous study on the same mice, we found increased liver TNF-α expression in response to the same combination (34), suggesting increased liver macrophage activities. Such upregulation of IL-10 does not seem to normalise liver metabolic markers, such as reduced triglyceride lipase and increased fatty acid synthase and collagen (34). Furthermore, the upregulated liver IL-10 did not increase serum IL-10 levels in the HFD+e-vapour group, which may not be the cause of suppressed immune response in the blood and adipose tissue. Therefore, there may be some unknown mechanisms that suppress the immune response, which requires further investigation (**Figure 5**).

One limitation of the study was that we did not examine the peripheral immune cell profile, which may also directly cause the serum cytokine changes in response to e-vapour exposure and/or the combination with HFD. It needs to be noted that in this study, the dose of e-vapour was relatively small, which follows the number of puffs using nicotine-containing e-fluid to model light smokers. However, this may well represent human users (eg. cloud chasers) who do not need to vape as frequently as those addicted to nicotine. However, future studies can assess if the pro-inflammatory effect of nicotine-free e-vapour is dose-dependent. In addition, we chose one time point for e-vapour exposure. It will be interesting to examine the change in serum cytokines and tissue gene expression in the acute and longer term setting, especially with a second insult, such as viral or bacterial infection. Another limitation is that we are unclear about what chemical(s) in the heated e-fluid induced systemic inflammatory response. PG/VG vehicle was only shown to be toxic at higher doses *in vitro*, which was enhanced by additional flavouring chemicals (35). In a rodent model, there was no evidence of toxicity induced by PG/VG aerosols at different concentrations compared with saline (36). Furthermore, additional chemicals other than flavouring may exist in the e-fluids (37), as the labels do not always accurately reflect the chemical composition in e-fluid products (38). The heating process causes chemical changes to occur, and the composition of the initial chemical mixture would affect this, even with one vial of e-liquid potentially being different from another (even from the same manufacturer). The biggest issue is that there are multiple devices, and some devices have vaporised at different temperatures. Nevertheless, future studies are still needed to analyse the chemical composition in the e-vapour to better understand which chemical(s) may be the key to inducing systematic inflammatory responses.

## Conclusion

In conclusion, sub-chronic nicotine-free e-vapour exposure potently induces a systemic inflammatory response, more so than longer-term exposure to an HFD.

## Data Availability Statement

The original contributions presented in the study are included in the article/**Supplementary Material**. Further inquiries can be directed to the corresponding author.

## Ethics Statement

The animal study was reviewed and approved by Animal Ethics and Care Committee at Northern Sydney Health District (RESP17/93).

## Author Contributions

HC, SS, and BO designed the study. HC, YC, and AT collected and analysed the samples. HC wrote the first draft. All authors contributed to the article and approved the submitted version.

## Funding

YC is supported by a Peter Doherty Fellowship fromt he National Health and Medical Research Council. The study is supported by a project grant (APP1158186) from the National Health and Medical Research Council Australia.

## Conflict of Interest

The authors declare that the research was conducted in the absence of any commercial or financial relationships that could be construed as a potential conflict of interest.

## Publisher’s Note

All claims expressed in this article are solely those of the authors and do not necessarily represent those of their affiliated organizations, or those of the publisher, the editors and the reviewers. Any product that may be evaluated in this article, or claim that may be made by its manufacturer, is not guaranteed or endorsed by the publisher.
