# Supplementary material for: Inhaled or Ingested, Which Is Worse, E-Vaping or High-Fat Diet?
Source: Front Immunol. 2022 Jun 15;13:913044. doi: 10.3389/fimmu.2022.913044 (PMC9240210; doi:10.3389/fimmu.2022.913044)
Supplement: Supplementary file 1 [file Table_1.docx]

**Inhaled or ingested, which is worse, e-vaping or high-fat diet?**

Hui Chen^a†^, Yik Lung Chan ^a,b†^, Andrew E Thorpe^a^, Carol A Pollock ^c^, Sonia Saad ^c^, Brian G Oliver ^a,b^ *

^a^ School of Life Sciences, Faculty of Science, University of Technology Sydney, NSW 2007, Australia

^b^ Respiratory Cellular and Molecular Biology, Woolcock Institute of Medical Research, The University of Sydney, NSW 2037, Australia

^c^ Kolling Institute of Medical Research, Royal North Shore Hospital, The University of Sydney, New South Wales 2065, Australia

† equal contribution

* Corresponding author:

Professor Brian G Oliver, School of Life Sciences, Faculty of Science, University of Technology Sydney; Respiratory Cellular and Molecular Biology, Woolcock Institute of Medical Research, The University of Sydney, Australia. [brian.oliver@uts.edu.au](mailto:brian.oliver@uts.edu.au)

**Supplementary Table 1. KiCqStart SYBR Green gene sequences for real-time PCR.**

| Gene | Forward primer sequence | Reverse primer sequence |
| --- | --- | --- |
| IFN-γ | TGAGTATTGCCAAGTTTGAG | CTTATTGGGACAATCTCTTCC |
| FRACTALKINE/CX3CL1 | CTTCCATTTGTGTACTCTGC | ACTCCTGGTTTAGCTGATAG |
| IL-10 | CAGGACTTTAAGGGTTACTTG | ATTTTCACAGGGGAGAAATC |
| MIP-3a/CCL20 | GCTATCATCTTTCACACGAAG | CATCTTCTTGACTCTTAGGC |
| MCP-5/CCL12 | ATTTTCACAGGGGAGAAATC | TGTGATCTTCAGGACCATAC |
| RANTES/CCL5 | AGGAGTATTTCTACACCAGC | CAGGGTCAGAATCAAGAAAC |
| β actin | GATGTATGAAGGCTTTGGTC | TGTGCACTTTTATTGGTCTC |

IFN-γ: Interferon gamma; FRACTALKINE/CX3CL1: C-X3-C motif chemokine ligand 1; IL-10: Interleukin-1; MIP-3a/CCL20: Macrophage Inflammatory Protein-3A/Chemokine (C-C Motif) ligand 20; MCP-5/CCL12: Monocyte chemoattractant proteins-5/ Chemokine (C-C Motif) ligand 12; RANTES/ CCL5: Regulated on Activation, Normal T Cell expressed and secreted/ Chemokine (C-C Motif) ligand 5.

**Supplementary Table 2. Serum levels of other cytokines measured**

| **Cytokines** | **Chow+sham**  **(pg/ml)** | **Chow+e-vapour**  **(pg/ml)** | **HFD+sham**  **(pg/ml)** | **HFD+e-vapour**  **(pg/ml)** |
| --- | --- | --- | --- | --- |
| TNF-α | 17.0±4.71 | 31.3±4.53* | 31.8±2.50* | 26.3±4.79 |
| MPI-1β | 1001±43 | 4751±946* | 3847±1144* | 3769±1086 |
| MIP-3α | 6.15±0.71 | 42.7±9.87** | 27.5±7.30* | 21.1±5.93 |
| ENA-78 | 75.0±26.7 | 227±52.6* | 102±37.0 | 153±47.8 |
| IL-4 | 0.23±0.029 | 0.98±0.21* | 0.73±0.21 | 0.71±0.18 |
| IP-10 | 27.6±4.09 | 167±40.6* | 114±35.4 | 97.7±30.2 |
| MCP-5 | 317±10.2 | 751±117** | 608±120 | 587±113 |
| MIP-3β | 67.5±13.4 | 127±23.5 | 140±26.9* | 138±20.0 |
| EOTAXIN-1 | 672±268 | 1409±490 | 1780±623 | 2917±1049 |
| EORAXIN-2 | 6642±1998 | 7897±1159 | 8691±1989 | 10397±1221 |
| I-309 | 9.03±2.24 | 15.9±3.39 | 15.2±2.98 | 15.4±1.70 |
| IL-1β | 58.1±12.6 | 69.8±11.4 | 81.9±8.48 | 113±27.9 |
| IL-16 | 67.2±9.94 | 64.1±11.5 | 60.1±9.56 | 54.1±7.37 |
| KC | 15.4±4.22 | 33.1±2.53 | 24.4±7.04 | 43.95±25.53 |
| MCP-3 | 18.3±1.45 | 23.86±1.41 | 29.8±5.75 | 51.5±26.7 |
| MDC | 19.9±11.1 | 29.6±11.3 | 46.0±13.8 | 98.7±45.8 |
| MIP-1 | 6.78±0.66 | 47.2±11.2 | 31.8±10.0 | 47.6±24.8 |
| TARC | 4.61±0.76 | 31.0±6.82 | 27.4±11.0 | 36.9±16.6 |
| SDF-1A | 534±239 | 791±398 | 5313±4214 | 6498±4859 |

Results are expressed as mean ± SEM, n=5. * P<0.05, ** P<0.01, compared to Chow+sham.

ENA: Epithelial neutrophil-activating peptide; EOTAXIN: eosinophil chemotactic protein; IL: Interleukin; I-309: Chemokine ligand 1; IP-10: Interferon gamma-induced protein 10; KC: keratinocytes-derived chemokine; MIP: macrophage inflammatory protein, MCP: monocyte chemotactic protein; MIP: macrophage inflammatory protein; MPI-1β: mannose phosphate isomerase; MDC: macrophage-derived chemokine; SDF: stroma cell-derived factor; TNF: tumour necrosis factor; TARC: thymus and activation regulated chemokine.
